# Creation of Reliable Relevance Judgments in Information Retrieval Systems Evaluation Experimentation through Crowdsourcing: A Review

**DOI:** 10.1155/2014/135641

**Published:** 2014-05-19

**Authors:** Parnia Samimi, Sri Devi Ravana

**Affiliations:** Department of Information Systems, Faculty of Computer Science and Information Technology, University of Malaya, 50603 Kuala Lumpur, Malaysia

## Abstract

Test collection is used to evaluate the information retrieval systems in laboratory-based evaluation experimentation. In a classic setting, generating relevance judgments involves human assessors and is a costly and time consuming task. Researchers and practitioners are still being challenged in performing reliable and low-cost evaluation of retrieval systems. Crowdsourcing as a novel method of data acquisition is broadly used in many research fields. It has been proven that crowdsourcing is an inexpensive and quick solution as well as a reliable alternative for creating relevance judgments. One of the crowdsourcing applications in IR is to judge relevancy of query document pair. In order to have a successful crowdsourcing experiment, the relevance judgment tasks should be designed precisely to emphasize quality control. This paper is intended to explore different factors that have an influence on the accuracy of relevance judgments accomplished by workers and how to intensify the reliability of judgments in crowdsourcing experiment.

## 1. Introduction

**Figure 1:**
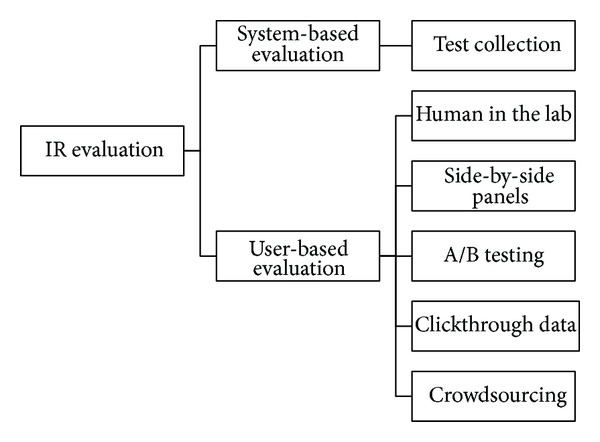
Classification of IR evaluation methods.

In order to have an effective Information Retrieval (IR) system and user satisfaction, evaluation of system performance is crucial. IR evaluation is to measure whether the system addresses the information needs of the users. There are two approaches for evaluating the effectiveness of IR systems as shown in Figure 1: (i) user-based evaluation and (ii) system-based evaluation [1]. In the system-based evaluation method, a number of assessors prepare a set of data which can be reused in later experiments. A common system-based evaluation approach is test collections, which is also referred to as the Cranfield experiments, which is the beginning of today's laboratory retrieval evaluation experiments [2]. The Text REtrieval Conference (TREC) was established in 1992 in order to support IR researches through providing the infrastructure for large scale evaluation of retrieval methodologies.

Test collection consists of three components: (i) document corpus which is a set of large size documents, (ii) topics which are a collection of search queries, and (iii) relevance judgments which involve human assessors appointed by TREC. The relevance judgment is partial since not all documents from the corpus have been judged by assessors because the judging task is not only costly but also time consuming. For instance, a complete judgment collection for TREC-2010 Web needs expert assessors to assess 1 billion documents. By assuming that an expert assessor can assess two documents in a minute, 347,000 days are needed for judging 1 billion documents. Therefore, to assess more documents, a large number of human experts need to be appointed which is costly [3].

**Figure 2:**
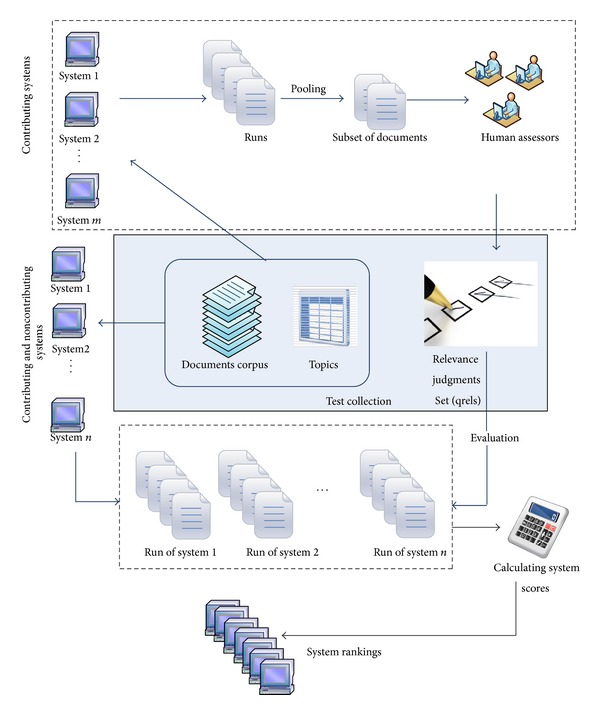
Typical IR evaluation process.

In TREC, pooling method has been used to recognize a subset of documents for judging.  Figure 2 shows the process of typical IR evaluation through test collection. The participating systems run their retrieval algorithms against the document corpus and topics in the test collection to generate a set of documents called runs. The systems 1, 2, …, *m* are the contributing systems for the pool creation and a collection of top ranked documents retrieved by contributing systems for each topic has been selected for judgment. The documents in the pool are judged by human assessors to create relevance judgment set, and all of the other documents outside the pool are considered as nonrelevant documents. Once the relevance judgments are ready, the whole set of runs retrieved by both contributing and noncontributing systems (1, 2, …, *n*) would be evaluated against relevance judgments or qrels to measure the accuracy and effectiveness of the retrieval systems through evaluation metrics. Each system gets a score for each topic and then scores are aggregated to achieve the overall performance score for a system. Finally, as a result of each IR experiment, the system ranking is generated for all of the systems. The major drawback of test collections is the huge cost of relevance assessment which is conducted by human experts. It needs different resources including time, infrastructure, and money while it does not scale up simply.

The user-based evaluation method quantifies the satisfaction of users by monitoring the user's interactions with the system.  Table 1 illustrates the user-based evaluation methods which are divided into five groups [4].

The drawback of human involvement in the user-based evaluation experiments in labs is the high cost of experimentation set up and the difficulty in repeating the experiment. In addition, this method limited to a small set of information needs accomplished by a small number of human subjects. The side-by-side panels just allow the comparison of two systems but it is not applicable to multiple systems. Using clickthrough data seems attractive because of the low cost of collecting data, but it needs a precise setup since it can be noisy. The term crowdsourcing was conceived by Howe based on Web 2.0 technology in a Wired Magazine article [5]. Recently, the use of the term “crowdsourcing” for relevance judgment is increasing to conquer the high cost that current evaluation methods have through expert judges. Running experiments within low cost and fast turnaround makes this approach very outstanding [4].

This paper discusses the issues related to using crowdsourcing for creating relevance judgment while giving advice on the implementation of successful crowdsourcing experiments and presents a state-of-the-art review on available methods to enhance the quality of the judgments in crowdsourcing experiments. The organization of this paper is as follows: the introductory section explains IR evaluation process and its different methods; Section 2 elaborates crowdsourcing and its application in different fields especially IR; Section 3 explains how accuracy of crowdsourcing results is measured; Section 4 highlights importance of task design in crowdsourcing as well as human features and monetary factors are explained in Section 5. The recently proposed methods on quality control of crowdsourcing will be reviewed in Section 6. The section on conclusion presents a look into the future on the use of crowdsourcing for IR evaluation and some suggestions for further study in related areas.

## 2. Introduction to Crowdsourcing 

Crowdsourcing is an upcoming research field for Information Systems scholars to create noteworthy contributions. It can be applied widely in various fields of computer science and other disciplines to test and evaluate studies as shown and explained in Table 2 [6].

**Figure 3:**
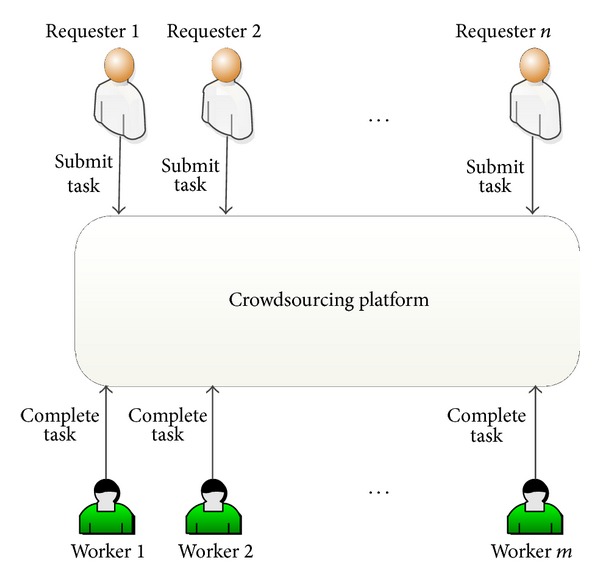
Crowdsourcing scheme.

There are various platforms for crowdsourcing such as Amazon Mechanical Turk (AMT) (https://www.mturk.com/mturk/welcome), Crowdflower [16], ODesk (https://www.odesk.com/), and Elance (https://www.elance.com/). Currently, AMT is the most popular and largest marketplace and crowdsourcing platform. It is a profit-oriented marketplace which allows requesters to submit tasks and workers to complete them. Human Intelligence Tasks (HIT) or microtasks is the unit of work to be accomplished.  Figure 3 presents the crowdsourcing scheme which includes multiple requesters who publish their tasks and workers who accomplish the tasks on a crowdsourcing platform.

**Figure 4:**
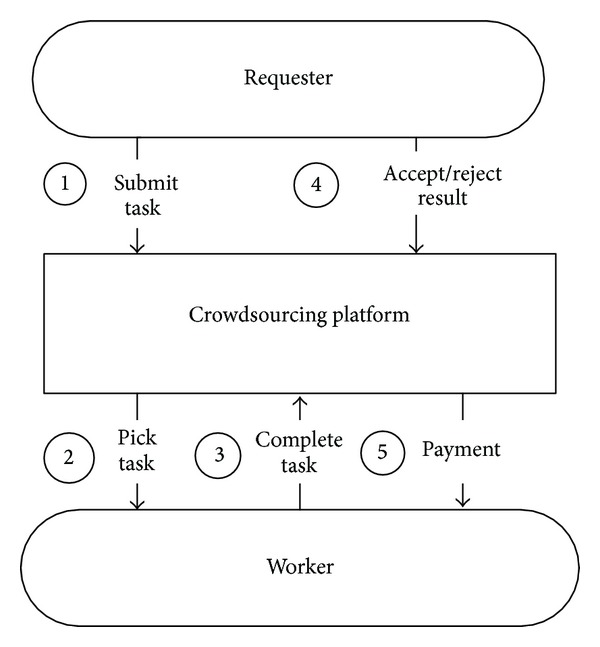
Flow of submitting and completing tasks via crowdsourcing.

Generally, the crowdsourcing process starts with publishing a task by requesters to the crowdsourcing platform. Workers select and work on a specific task of their interest and complete it. The requesters assess the results of the tasks done by workers. If the results are acceptable to requesters, they would pay the workers. Otherwise the workers might be rejected because of performing the task carelessly. The flow of submitting and completing tasks via crowdsourcing is shown in Figure 4.

Simplicity is the main feature of crowdsourcing. Crowdsourcing platforms enable the requesters to have fast access to an on-demand, global, scalable workforce and the workers to choose thousands of tasks to accomplish online. In AMT, the requesters are able to create HITs via application programming interface (API) which enable requesters to distribute tasks pragrammatically or use template via dashboard. Crowdsourcing platforms were also suggested for data collection as a viable choice [17]. Mason and Suri [18] stated three advantages of crowdsourcing platforms: (i) allowing a large number of workers to take part in experiments with low payment, (ii) workers are from diverse language, culture, background, age, and country, and (iii) low cost at which the researches can be carried on. Precise design and quality control are required to optimize work quality and conduct a successful crowdsourcing experiment. In general, there are three objectives that most researchers draw attention to in this field of research especially in IR evaluation which are (i) to determine the agreement percentage between experts and crowdsourcing workers, (ii) to find out factors that have an effect on the accuracy of the workers' results, and (iii) to find the approaches to maximize the quality of workers' results. A survey of crowdsourcing was done in terms of social, academic, and enterprise [19] while assessing different factors which cause motivation and participation of volunteers including the following:

- role-oriented crowdsourcing (leadership and ownership),
- behaviour-oriented crowdsourcing (attribution, coordination, and conflict),
- media-oriented crowdsourcing (mobile computing and ubiquitous computing).

However, this study looks from the academic aspect of crowdsourcing specifically focusing on factors that contribute to a successful and efficient crowdsourcing experiment in the area of information retrieval evaluation.

## 3. Accuracy and Reliability of Relevance Judgments in Crowdsourcing

The use of crowdsourcing in creating relevance judgment for an IR evaluation is generally validated through measuring the agreement between crowdsourcing workers and human assessors. This is to see whether crowdsourcing is a reliable replacement for human assessors. In crowdsourcing experiments, the interrater or interannotator agreement is used to measure the performance and to analyse the agreement between crowdsourcing workers and human assessors. The score of homogeneity in the rating list given by judges is the interrater agreement. Various statistical methods are used to calculate the interrater agreement.  Table 3 summarizes four common methods suggested to calculate the interrater agreement between crowdsourcing workers and human assessors for relevance judgment in IR evaluation [20].

Alonso et al. [15], pioneers of crowdsourcing in IR evaluation, applied crowdsourcing through Amazon Mechanical Turk on TREC data. Their results showed that crowdsourcing is a low cost, reliable, and quick solution and could be considered an alternative for creating relevance judgment by experts but it is not a replacement for current methods. This is due to the fact that there are still several gaps and questions that should be addressed by future researches. The scalability of crowdsourcing approach in IR evaluation has not been investigated yet [20].

## 4. Experimental Design in Crowdsourcing

Beyond the workers' attention, culture, preferences, and skills, the presentation and properties of HITs have an influence on the quality of the crowdsourcing experiment. The careful and precise task design, rather than filtering the poor quality work after accomplishing the task, is an important task since the complicated tasks distract cheaters [24]. It is possible that qualified workers accomplish erroneous tasks if the user interface, instructions, and design have a poor quality. Different phases in the implementation of crowdsourcing experiment are similar to software development. The details of each phase are identified for creating relevance judgment task as follows.

*Planning*

- Choosing a crowdsourcing platform.
- Defining a task.
- Preparing a dataset.

*Design and Development*

- Selecting topics.
- Choosing a few documents for each topic (relevant and nonrelevant).
- Task definition, description, and preparation.

*Verifying*

- Debugging by using internal expert team.
- Take note of shortcoming of the experiment.
- Using external inexpert to test.
- Reviewing and clearing the instruction.
- Comparing the results of experts with inexpert teams.

*Publishing*

- Monitoring/work quality.

The impact of the two methods of HIT design, the full and simple design, was assessed on the accuracy of labels [25]. The full design method prequalifies and restricts workers while the simple design method includes less quality control setting and restriction on the workers. However, the output of the full design method has a higher quality. In general, crowdsourcing is a useful solution for relevance assessment if the tasks are designed carefully and the methods for aggregating labels are selected appropriately. Experiment design is discussed based on three categories: (i) task definition, description, and preparation, (ii) interface design, and (iii) granularity.

### 4.1. Task Definition, Description, and Preparation

The information that is presented to the workers about crowdsourcing task is task definition. An important issue of implementing a crowdsourcing experiment is task description that is a part of task preparation. Clear instruction which is a part of task description is crucial to have a quick result usually in less than 24 hours. All workers should have a common understanding about the task and the words used must be understandable by different workers [26]. Task description should consider the characteristics of workers such as language or expertise [27]. For example, to avoid jargon, plain English is suggested since the population is diverse [28]. As creating relevance judgments requires reading text, using plain English and simple words do help in a successful experiment, having the phrase “I do not know” was suggested in response as it allows workers to convey that they do not have sufficient knowledge to answer the question [26]. Getting user feedback at the end of the task by asking an open-ended question is recommended to improve the quality and avoid spammers. This kind of question can be optional. If the answer is useful, the requester can pay bonus [28]. In creating relevance judgments, one has to read text; hence instructions and questions should be readable, clear, and presented with examples. An example of relevance judgment task is shown below.

*Relevance Judgment*

*Instruction*. Evaluate the relevance of a document to the given query.

*Task*. Please evaluate the relevance of the following document about* Alzheimer*.

“*Dementia is a loss of brain function that occurs with certain diseases. Alzheimer's disease (AD), is one form of dementia that gradually gets worse over time. It affects memory, thinking, and behavior.*”

Please rate the above document according to its relevance to* Alzheimer* as follows:

- Highly relevant
- Relevant
- Not relevant

Please justify your answer: — — — — — —

### 4.2. Interface Design

Users or workers access and contribute to the tasks via user interface. Three design recommendations were suggested to have a doing well task completion along with paying less; they are designing a user interface and instructions understandable by novice users, translating the instruction to the local language as well as English, and preparing a video tutorial [29]. It also suggested the use of colours, highlights, bold, italics, and typefaces to enhance comprehensibility [26]. Verifiable questions such as common-knowledge questions within the task are also useful to validate the workers. It leads to a reduction in the number of worthless results since the users are aware that their answers are being analysed [30]. Another research work showed that more workers may be attracted to the user friendly and simple interface, and the quality of outcome may increase while reliable workers may be unenthusiastic about the unreasonably complex user interface which leads to delay [27].

### 4.3. Granularity

Tasks can be divided into three broad groups: routine, complex, and creative tasks. The tasks which do not need specific expertise are called routine such as creating relevance judgment. Complex tasks need some general skills like rewriting a given text. On the other hand, creative tasks need specific skill and expertise such as research and development [31]. Submitting small tasks by splitting the long and complex tasks was recommended since small tasks tend to attract more workers [26]. However, some studies showed that novel tasks that need creativity tend to distract the cheaters and attract more qualified and reliable workers [24, 32]. In a separate study, Difallah et al. [33] stated that designing complicated tasks is a solution to filter out the cheaters. On the other hand, money is the main reason why most of the workers accomplish the tasks. However, the workers who do the task because of the prospect of becoming a celebrity and fun are the least truthful while the workers who are provoked by fulfillment and fortune are the most truthful workers. Eickhoff and de Vries [24] found that the workers who do the job for entertainment, not for money, rarely cheat. So, designing and representing HITs in an exciting and entertaining way lead to better results along with cost efficiency.

## 5. Human Features and Monetary Factors in Crowdsourcing 

Effect of human features like knowledge and monetary factors such as payment on accuracy and success of crowdsourcing experiments is discussed in this section. The discussion is based on three factors, namely, (i) worker profile in crowdsourcing, (ii) payment in crowdsourcing, and (iii) compensation policy in crowdsourcing.

### 5.1. Worker Profile in Crowdsourcing

One of the factors that have an influence on the quality of results is worker profile. Worker profile consists of reputation and expertise. Requesters' feedback about workers' accomplishments creates reputation scores in the systems [34]. Requesters also should maintain a positive reputation since workers may refuse accepting HIT from poor requesters [17]. Expertise can be recognized by credentials and experience. Information such as language, location, and academic degree is credentials while experience refers to knowledge that worker achieves through crowdsourcing system [27]. Location is another important factor that has a strong effect on the accuracy of the results. A study conducted by Kazai et al. [35] indicated that Asian workers performed poor quality work compared with American or European workers. The Asians mostly preferred simple design of HIT while American workers preferred full design. In crowdsourcing platforms such as AMT and Crowdflower, it is possible to limit the workers to the specific country while designing tasks. However, creating relevance judgment is a routine task and does not need specific expertise or experience.

### 5.2. Payment in Crowdsourcing

Payment impacts on accuracy of the results in crowdsourcing since the workers who are satisfied with the payment accomplish the tasks more accurately than the workers who are not contented [32]. Monetary or nonmonetary reasons can be the motivation for the workers of crowdsourcing platforms [36]. A study conducted by Ross et al. [37] to find the motivation of the workers in crowdsourcing showed that money is the main incentive of 13% of Indian and 5% of US workers. Another study carried out by Ipeirotis [38] reported that AMT was the main income of 27% of Indians and 12% of US workers. Kazai [39] stated that higher payment leads to better quality of work while Potthast et al. [40] reported that higher payment only has an effect on completion time rather than on the quality of results.

Kazai et al. [32] analysed the effect of higher payment to the workers on accuracy of the results. Although higher payment encourages more qualified workers in contrast with lower payment which upsurges the sloppy workers, sometimes unethical workers are attracted by higher payment. In this case, strong quality control method can prevent poor result as well as filtering. Other studies reported that by increasing payment, it leads to increase in quantity rather than quality while some studies showed that considering greater payment may only influence getting the task done faster but not better as increasing payment incentive speeds up work [41, 42]. However, a reasonable pay is a better cautious solution as high pay tasks attract spammers as well as legitimate workers [43]. Indeed, the payment amount should be fair and based on the complexity of the tasks.

Payment method is another factor which impacts on the quality of the outputs of the workers [41, 44], for instance, a task which requires finding 10 words in a puzzle; more puzzles would be solved with method of payment per puzzle rather than payment per word [45]. It is better to pay workers after the work quality has been confirmed by measuring the accuracy and clarification that payment is based on the quality of the completed work. This can help in achieving better results from workers [26]. In an experiment conducted by Alonso and Mizzaro [20], the workers were paid $0.02 for relevancy evaluation that took about one minute consisting of judging the relevancy of one topic and document. In another experiment, the payment was $0.04 for judging one topic against ten documents [46].

### 5.3. Compensation in Crowdsourcing

A proper compensation policy and inducement has an impact on the quality of the results [47, 48]. There are two types of incentives, extrinsic incentives like monetary bonus such as extra payment and intrinsic incentives such as personal enthusiasm [27]. Rewards were categorized into psychological, monetary, and material [47]. Mason and Watts [41] found that using nonfinancial compensation to motivate workers such as enjoyable tasks or social rewards is a better choice than financial rewards and have more effect on quality. It was also reported that using social rewards is like harnessing intrinsic motivation to improve the quality of work [49]. The use of intrinsic along with extrinsic incentives was recommended to motivate more reliable workers to perform a task. Furthermore, considering the task requirements, properties, and social factors such as income and interests of workers according to the profiles of requesters and workers is vital in selecting proper compensation policy [27]. Offering bonus for writing comments and justification or paying bonus to the qualified and good workers who perform the tasks accurately and precisely was recommended for relevance evaluation task [26].

In addition to task design, human features and monetary factors are important to have a successful crowdsourcing experiment; quality control is another vital part of crowdsourcing experiment which will be elaborated on in the following section.

## 6. Quality Control in Crowdsourcing

Managing the workers' act is a crucial part of crowdsourcing because of the sloppy workers who accomplish the tasks carelessly or spammers who trick and complete the tasks inaccurately. Different worker types by using different factors including label accuracy, HIT completion time, and fraction of useful labels were determined through behavioral observation [50] as explained below (see Table 4). This can help in developing methods for HIT design to attract the proper workers for the job.  Table 5 shows different types of workers classified based on their average precision [51].

In 2012, to detect random spammers, for each worker *w*, Vuurens and de Vries [52] calculated the RandomSpam score as the average squared ordinal distance (it shows how the judgment of one worker is different from the other worker), *ord*_*ij*_^2^ between their judgments *j* ∈ *J*_*w*_, and judgments by other workers on the same query-document pair *i* ∈ *J*_*j*,*w*_ as shown below:

(1) $$\begin{array}{c} {\text{RandomSpam}}_{w}=\frac{\sum_{j\in J_{w}}^{}\sum_{i\in J_{j,\overset{-}{w}}}^{}{ord}_{ij}^{2}}{\sum_{j\in J_{w}}^{}\left|J_{j,\overset{-}{w}}\right|}. \end{array}$$

In addition, to detect the uniform spammers, the* UniformSpam* score is calculated as shown below:

(2) UniformSpamw  =∑s∈S|S|·(fs,Jw−1)·(∑j∈Js,w∑i∈Jj,w−disagreeij)2∑s∈S∑j∈Js,w|Jj,w−|,

where disagree_*ij*_ is the number of disagreements between judgments *J*_*s*,*w*_ which happen within label sequence *s* and judgments *J*_*j*,*w*_ and *S* is a set of all possible label sequences *s* with length |*s* | = 2 or 3, while *f*_*s*,*J*_*w*__ is the frequency at which label sequence *s* occurs within worker *w*'s time-ordered judgments *J*_*w*_.

Quality control in crowdsourcing area is defined as the extent to which the provided outcome fulfills the requirements of the requester. Quality control approaches can be categorized into (i) design-time approaches which were employed while the requester prepared the task before submission and (ii) run-time approaches which were used during or after doing the tasks. Another classification for quality control methods is (i) filtering workers and (ii) aggregating labels that are discussed in this section [53]. In other words, filtering workers are design-time approaches and aggregating labels methods can be considered as run-time approaches [27].

### 6.1. Design-Time Methods

There are three approaches to select workers: (i) credential-based, (ii) reputation-based, and (iii) open-to-all. It is also possible to use the combination of the three approaches. Credential-based approach is used in systems where users are well profiled. However, this approach is not practical in crowdsourcing systems since users are usually unknown. Reputation-based approach selects workers based on their reputation like AMT by using approval rate parameter. Open-to-all approach such as in Wikipedia allows any worker to contribute and it is easy to use and implement but increases the number of unreliable workers [27]. Requesters are also able to implement their own qualification methods [18] or combine different methods. In total, there are various methods of filtering workers which identify the sloppy workers at first and then exclude them. We have categorized the methods into five categories.  Table 6 presents a list of these methods.

When using qualification test, it is still possible that workers perform tasks carelessly. Moreover, there are two issues that researchers encounter with qualification test: (i) tasks with qualification test may take longer to complete as most workers prefer tasks without qualification test and (ii) the cost of developing and maintaining the test continuously. Honey pots or gold standard is used when the results are clear and predefined [54]. The honey pots are faster than qualification test as it is easy and quick to identify workers who answer the questions at random. Combining qualification test and honey pots is also possible for quality control [26].

Qualification settings such as filtering workers especially based on origin have an important impact on the cheater rates. For instance, some assume that workers from developed countries have lesser cheater rates [24]. Setting approval rate has been used by AMT. AMT provides a metric called the approval rate to prefilter the workers. It represents percentage of assignments the worker has performed and confirmed by the requester over all tasks the worker has done. Generally, it is the total rating of each worker in the system. It is also possible to limit the tasks to the master workers. In AMT, the group of people who accomplish HITs with a high degree of accuracy across a variety of requesters are called master workers. Master workers expect to receive higher payment. Limiting the HITs to the master workers gets better quality results [32]. If the high approval rate is considered for quality control, it would take a longer time to complete the experiment since the worker population may be decreasing [28]. In general, the setting should be done according to the task type. If the task is complicated, the approval rate and other settings should be set precisely or the task is assigned to master workers only. If the task is routine, ordinary workers with simple setting can be effective. Trap questions are also used to detect careless workers who do not read the instructions of the task carefully. Kazai et al. [25] designed two trap questions to avoid this situation: “Please tick here if you did NOT read the instructions” and at each page “I did not pay attention.” All of the unreliable workers may not be detected by trap questions but it showed strange behavior and it can be effective both in discouraging and identifying spammers. In crowdsourcing, there are some programs or bots designed to accomplish HITs automatically [55] and these kinds of data definitely are of poor quality [18]. CAPTCHAs and reCAPTCHA are used in crowdsourcing experiment to detect superficial workers and random clicking on answers [29, 32]. These methods are easy to use and inexpensive.

It is also possible to use a combination of the above methods to filter workers. For example, a real time strategy applying different methods to filter workers was proposed in recruiting workers and monitoring the quality of their works. At first, a qualification test was used to filter the sloppy workers and the completion time of HITs was calculated to reflect on the truthfulness of the workers. Then, a set of gold questions were used to evaluate the skills of workers [56]. However, by using these methods to filter workers, there is the possibility of lesser quality being recruited to do a work. The quality control methods used after filtering workers in run time are explained in the subsequent section.

### 6.2. Run-Time Methods

Although design-time techniques can intensify quality, there is the possibility of low quality because of misunderstanding while doing the tasks. Therefore, run-time techniques are vital to high quality results. Indeed, quality of the results would be increased by applying both approaches [27]. In crowdsourcing, if we assume that one judgment per each example or task is called single labeling method, the time and cost may be saved. However, the quality of work is dependent on an individual's knowledge, and in order to solve this issue of single labeling methods, integrating the labels from multiple workers was introduced [61, 62]. If labels are noisy, multiple labels can be desirable to single labeling even in the former setting when labels are not particularly low cost [63]. It leads to having more accuracy for relevance judgments [64]. An important issue related to multiple labels per example is how to aggregate labels accurately and effieciently from various workers into a single consensus label. Run-time quality control methods are explained in Table 7.

The MV is a better choice for routine tasks which are paid a lower payment since it is easy to implement and achieve reasonable results depending on truthfulness of the workers [53]. The drawback of this method is that the consensus label is measured for a specific task without considering the accuracy of the workers in other tasks. Another drawback of this method is that MV considers all workers are equally good. For example, if there is only one expert and the others are novices and the novices give the same inaccurate responses, the MV considers the novices' answer as the correct answer because they are the majority.

In EM algorithm, a set of estimated accurate answers for each task and a set of matrixes that include the list of workers errors (in that the replies might not be the corrected answers) are the outputs. The error rate of each worker can be accessed by this confusion matrix. However, to measure the quality of a worker, the error rate is not adequate since workers may have completed the task carefully but with bias. For example, in labeling websites, parents with young children are more conservative in classifying the websites. To prevent this situation, a single scalar score can be assigned to each worker corresponding to the completed labels. The scores lead to separation of error rate from worker bias and satisfactory treatment of the workers. Approximately five labels are needed for each example for this algorithm for it to become accurate [65]. Recently Carpenter [66] and Raykar et al. [67] proposed a Bayesian version of the EM algorithm by using confusion matrix. A probabilistic framework was proposed by Raykar et al. [67] in the case of no gold standard with multiple labels. A specific gold standard is created repeatedly by the proposed algorithm and the performances of workers are measured and then the gold standard is refined according to the performance measurements. Hosseini et al. [64] evaluated the performance of EM and MV for aggregating labels in relevance assessment. His experiment results showed that the EM method is a better solution to problems related to the reliability of IR system ranking and relevance judgment especially in the case of small number of labels and noisy judgments. Another study compared MV and EM methods which stated that EM outperformed MV when 60% of workers were spammers [51]. However, a combination of these two methods could produce more accurate relevance judgments.

Considering the time taken to complete the task is an example of observation of the pattern of responses which was used to determine random answers of unreliable workers [30]. As tasks that were completed fast deemed to have poor quality, completion time is a robust method of detecting the sloppy workers. Soleymani and Larson [75] used crowdsourcing for affective annotation of video in order to improve the performance of multimedia retrieval systems. The completion time of each HIT was compared to video length in order to evaluate quality. In another study, the time that each worker spent on judgment was assessed in order to control quality [58]. Three types of patterns were found: (i) normal pattern whereby the workers begin slowly and get faster when they learn about the task, (ii) periodic pattern, a peculiar behavior since some of the judgments are done fast and some slow, and (iii) interrupted pattern, which refers to interruption in the middle of doing tasks. This method in combination with other methods of quality control can be effective in crowdsourcing experiments since this method alone may not be able to detect all sloppy workers. Another method of quality control, expert review, is commonly applied in practice, but the drawbacks of this method are the high cost of employing experts and being time consuming. This approach is not applicable in IR evaluation since crowdsourcing is used to lower the cost of hiring experts for creating relevance judgment. Therefore, if the expert review method is used to aggregate judgments, the cost would be increased.

The drawbacks of using crowdsourcing to create relevance judgment are (i) each worker judges a small number of examples; and (ii) to decrease cost, few judgments are collected per example. Therefore, the judgments are imbalanced and sparse as each worker assesses a small number of examples. The MV is vulnerable to this problem while EM indirectly addresses this problem and PMF tackles this issue directly [70].

Different quality control methods applied in crowdsourcing experiment in different areas of the study were listed and discussed both in design-time approaches and run-time approaches. Although successful experiments are crucial to both approaches, the important point is that quality control method should be well-suited to crowdsourcing platform.

## 7. Conclusion and Future Work

Crowdsourcing is a novel phenomenon being applied to various fields of studies. One of the applications of the crowdsourcing is in IR evaluation experiments. Several recent studies show that crowdsourcing is a viable alternative to the creation of relevance judgment; however, it needs precise design and appropriate quality control methods to be certain about the outcomes. This is particularly imperative since the workers of crowdsourcing platforms come from diverse cultural and linguistic backgrounds.

One of the important concerns in crowdsourcing experiments is the possibility of untrustworthiness of workers trying to earn money without paying due attention to their tasks. Confusion or misunderstanding in crowdsourcing experiments is another concern that may happen due to low quality in the experimental designs. Conclusively, the quality control in crowdsourcing experiments is not only a vital element in the designing phase but also in run-time phase. The quality control and monitoring strategies should be considered and examined during all stages of the experiments. Moreover, the user interface and instruction should be comprehensible, motivating, and exciting to the users. The payment should be reasonable but monetary or nonmonetary rewards can be also considerable. Although there are efforts in developing crowdsourcing experiments in IR area in recent years, there are several limitations (some are listed below) pending to refine the various approaches:

- currently, a random topic-document is shown to the workers to judge; workers should be provided with the freedom to choose their topic of interest for relevance judgment,
- to scale up creating relevance judgments for a larger set of topic-document to find out whether crowdsourcing is a replacement for hired assessors in terms of creating relevance judgments,
- to design a more precise grading system for workers rather than approval rate where requesters can find out each workers' grade in each task type. For example, the requesters are able to know which workers have a good grade in creating relevance judgments,
- to access reliable personal information of workers such as expertise, language, and background by requesters to decide which type of workers are allowed to accomplish the tasks,
- trying different aggregating methods in creating relevance judgments and evaluating the influence on correlation of judgments with TREC experts.

Crowdsourcing is an exciting research area with several puzzles and challenges that require further researches and investigations. This review shed a light on some aspects of using crowdsourcing in IR evaluation with insight into issues related to crowdsourcing experiments in some of the main stages such as design, implementation, and monitoring. Hence, further research and development should be conducted to enhance the reliability and accuracy of relevance judgments in IR experimentation.

## Figures and Tables

**Table 1:** User-based evaluation methods.

User-based methods	Description
Human in the lab	This method involves human experimentation in the lab to evaluate the user-system interaction

Side-by-side panels	This method is defined as collecting the top ranked answers generated by two IR systems for the same search query and representing them side by side to the users. To evaluate this method, in the eyes of human assessor, a simple judgment is needed to see which side retrieves better results

A/B testing	A/B testing involves numbers of preselected users of a website to analyse their reactions to the specific modification to see whether the change is positive or negative

Using clickthrough data	Clickthrough data is used to observe how frequently users click on retrieved documents for a given query

Crowdsourcing	Crowdsourcing is defined as outsourcing tasks, which was formerly accomplished inside a company or institution by employees assigned externally to huge, heterogeneous mass of potential workers in the form of an open call through Internet

**Table 2:** Different applications of crowdsourcing.

Application	Description
Natural language processing	Crowdsourcing technology was used to investigate linguistic theory and language processing [7]

Machine learning	Automatic translation by using active learning and crowdsourcing was suggested to reduce the cost of language experts [8, 9]

Software engineering	The use of crowdsourcing was investigated to solve the problem of recruiting the right type and number of subjects to evaluate a software engineering technique [10]

Network event monitoring	Using crowdsourcing to detect, isolate, and report service-level network events was explored which was called CEM (crowdsourcing event monitoring) [11]

Sentiment classification	The issues in training a sentiment analysis system using data collected through crowdsourcing were analysed [12]

Cataloguing	The application of crowdsourcing for libraries and archives was assessed [13]

Transportation plan	Use of crowdsourcing was argued to enable the citizen participation process in public planning projects [14]

Information retrieval	To create relevance judgments, crowdsourcing was suggested as a feasible alternative [15]

**Table 3:** Statistics for calculating the interrater agreement.

Methods	Description
Joint-probability of agreement (percentage agreement) [20]	The simplest and easiest measure based on dividing number of times for each rating (e.g.,1,2,…, 5), assigned by each assessor, by the total number of the ratings

Cohen's kappa [21]	A statistical measure to calculate interrater agreement among raters. This measurement is more robust than percentage agreement since this method considers the effects of random agreement between two assessors

Fleiss' kappa [22]	An extended version of Cohen's kappa. This measurement considers the agreement among any number of raters (not only two)

Krippendorff's alpha [23]	The measurement is based on the overall distribution of assessors regardless of which assessors produced the judgments

**Table 4:** Worker types based on their behavioral observation.

Workers	Description
Diligent	Completed tasks precisely with a high accuracy and longer time spent on tasks
Competent	Skilled workers with high accuracy and fast work
Sloppy	Completed tasks quickly without considering quality
Incompetent	Completed tasks in a time with low accuracy
Spammer	Did not deliver useful works

**Table 5:** Worker types based on their average precision.

Workers	Description
Proper	Completed tasks precisely
Random spammer	Gave a worthless answer
Semirandom spammer	Answered incorrectly on most questions while answering correctly on few questions, hoping to avoid detection as a spammer
Uniform spammer	Repeated answers
Sloppy	Not precise enough in their judgments

**Table 6:** Design-time methods.

Method	Description	Platform	Example
Qualification test	Qualification test is a set of questions which the workers must answer to qualify for doing the tasks	AMT	IQ test [57]

Honey pots or gold standard data	Creating predefined questions with known answers is honey pots [54]. If the workers answer these questions correctly, they are marked as appropriate for this task	Crowdflower [16]	—

Qualification settings	Some qualification settings are set when creating HITs	AMT, Crowdflower	Using approval rate

Trap questions	This method is about designing HITs along with a set of questions with known answers to find unreliable workers [58]	—	—

CAPTCHAs and reCAPTCHA	CAPTCHAs are an antispamming technique to separate computers and humans to filter automatic answers. A text of the scanned page is used to identify whether human inputs data or spam is trying to trick the web application as only a human can go through the test [59]. reCAPTCHA is the development of CAPTCHAs [60]	—	—

**Table 7:** Run-time methods.

Method	Description
Majority voting (MV)	MV is a straightforward and common method which eliminates the wrong results by using the majority decision [31, 61, 68]

Expectation maximization (EM) algorithm	EM algorithm measures the worker quality by estimating the accurate answer for each task through labels completed by different workers using maximum likelihood. The algorithm has two phases: (i) the correct answer is estimated for each task through multiple labels submitted by different workers, accounting for the quality of each worker and (ii) comparing the assigned responses to the concluded accurate answer in order to estimate quality of each worker [69]

Naive Bayes (NB)	Following EM, NB is a method to model the biases and reliability of single workers and correct them in order to intensify the quality of the workers' results. According to gold standard data, a small amount of training data labeled by expert was used to correct the individual biases of workers. The idea is to recalibrate answers of workers to be more matched with experts. An average of four inexpert labels for each example is needed to emulate expert level label quality. This idea helps to improve annotation quality [68]

Observation of the pattern of responses	Looking at the pattern of answers is another effective way of filtering unreliable responses as some untrustworthy workers have a regular pattern, for example, selecting the first choice of every question

Probabilistic matrix factorization (PMF)	Using probabilistic matrix factorization (PMF) that induces a latent feature vector for each worker and example to infer unobserved worker assessments for all examples [70]. PMF is a standard method in collaborative filtering through converting crowdsourcing data to collaborative filtering data to predict unlabeled labels from workers [71, 72]

Expert review	Expert review uses experts to evaluate workers [73]

Contributor evaluation	The workers are evaluated according to quality factors such as their reputation, experience, or credentials. If the workers have enough quality factors, the requester would accept their tasks. For instance, Wikipedia would accept the article written by administrators without evaluation [27]. For instance, the tasks that are submitted by the workers who have a higher approval rate or master workers would be accepted without doubt

Real-time support	The requesters give workers feedback about the quality of their work in real time while workers are accomplishing the task. This helps workers to amend their works and the results showed that self-assessment and external feedback improve the quality of the task [48]. Another real-time approach was proposed by Kulkarni et al. [74] where requesters can follow the workers workflows while solving the tasks. A tool called Turkomatic was presented which employees workers to do tasks for requesters. While workers are doing the task, requesters are able to monitor the process and view the status of the task in real time

## References

[1] Voorhees EM (2002). The philosophy of information retrieval evaluation. *Evaluation of Cross-Language Information Retrieval Systems*.

[2] Cleverdon C (1967). The Cranfield tests on index language devices. *Aslib Proceedings*.

[3] Moghadasi SI, Ravana SD, Raman SN (2013). Low-cost evaluation techniques for information retrieval systems: a review. *Journal of Informetrics*.

[4] Baeza-Yates R, Ribeiro-Neto B (2011). *Modern Information Retrieval: the Concepts and Technology Behind Search*.

[5] Howe J (2006). The rise of crowdsourcing. *Wired Magazine*.

[6] Zhao Y, Zhu Q (2012). Evaluation on crowdsourcing research: current status and future direction. *Information Systems Frontiers*.

[7] Munro R, Bethard S, Kuperman V Crowdsourcing and language studies: the new generation of linguistic data.

[8] Ambati V, Vogel S, Carbonell J (2010). Active learning and crowd-sourcing for machine translation. *Language Resources and Evaluation (LREC)*.

[9] Callison-Burch C Fast, cheap, and creative: evaluating translation quality using amazon’s mechanical turk.

[10] Stolee KT, Elbaum S Exploring the use of crowdsourcing to support empirical studies in software engineering.

[11] Choffnes DR, Bustamante FE, Ge Z Crowdsourcing service-level network event monitoring.

[12] Brew A, Greene D, Cunningham P Using crowdsourcing and active learning to track sentiment in online media.

[13] Holley R Crowdsourcing and social engagement: potential, power and freedom for libraries and users.

[14] Brabham DC (2009). Crowdsourcing the public participation process for planning projects. *Planning Theory*.

[15] Alonso O, Rose DE, Stewart B (2008). Crowdsourcing for relevance evaluation. *ACM SIGIR Forum*.

[16] https://http://www.crowdflower.com/.

[17] Paolacci G, Chandler J, Ipeirotis PG (2010). Running experiments on amazon mechanical turk. *Judgment and Decision Making*.

[18] Mason W, Suri S (2012). Conducting behavioral research on amazon’s mechanical turk. *Behavior Research Methods*.

[19] Pan Y, Blevis E A survey of crowdsourcing as a means of collaboration and the implications of crowdsourcing for interaction design.

[20] Alonso O, Mizzaro S (2012). Using crowdsourcing for TREC relevance assessment. *Information Processing and Management*.

[21] Cohen J (1960). A coefficient of agreement for nominal scales. *Educational and Psychological Measurement*.

[22] Fleiss JL (1971). Measuring nominal scale agreement among many raters. *Psychological Bulletin*.

[23] Krippendorff K (1970). Estimating the reliability, systematic error and random error of interval data. *Educational and Psychological Measurement*.

[24] Eickhoff C, de Vries AP (2013). Increasing cheat robustness of crowdsourcing tasks. *Information Retrieval*.

[25] Kazai G, Kamps J, Koolen M, Milic-Frayling N Crowdsourcing for book search evaluation: Impact of HIT design on comparative system ranking.

[26] Alonso O (2013). Implementing crowdsourcing-based relevance experimentation: an industrial perspective. *Information Retrieval*.

[27] Allahbakhsh M, Benatallah B, Ignjatovic A, Motahari-Nezhad HR, Bertino E, Dustdar S (2013). Quality control in crowdsourcing systems: issues and directions. *IEEE Internet Computing*.

[28] Alonso O, Baeza-Yates R (2011). Design and implementation of relevance assessments using crowdsourcing. *Advances in Information Retrieval*.

[29] Khanna S, Ratan A, Davis J, Thies W Evaluating and improving the usability of mechanical turk for low-income workers in India.

[30] Kittur A, Chi EH, Suh B Crowdsourcing user studies with mechanical turk.

[31] Hirth M, Hoßfeld T, Tran-Gia P (2013). Analyzing costs and accuracy of validation mechanisms for crowdsourcing platforms. *Mathematical and Computer Modelling*.

[32] Kazai G, Kamps J, Milic-Frayling N (2013). An analysis of human factors and label accuracy in crowdsourcing relevance judgments. *Information Retrieval*.

[33] Difallah DE, Demartini G, Cudré-Mauroux P Mechanical cheat: spamming schemes and adversarial techniques on crowdsourcing platforms.

[34] De Alfaro L, Kulshreshtha A, Pye I, Adler BT (2011). Reputation systems for open collaboration. *Communications of the ACM*.

[35] Kazai G, Kamps J, Milic-Frayling N The face of quality in crowdsourcing relevance labels: demographics, personality and labeling accuracy.

[36] Hammon L, Hippner H (2012). Crowdsourcing. *Wirtschaftsinf*.

[37] Ross J, Irani L, Silberman MS, Zaldivar A, Tomlinson B Who are the crowdworkers? Shifting demographics in mechanical turk.

[38] Ipeirotis P Demographics of mechanical turk.

[39] Kazai G (2011). In search of quality in crowdsourcing for search engine evaluation. *Advances in Information Retrieval*.

[40] Potthast M, Stein B, Barrón-Cedeño A, Rosso P An evaluation framework for plagiarism detection.

[41] Mason W, Watts DJ (2010). Financial incentives and the performance of crowds. *ACM SIGKDD Explorations Newsletter*.

[42] Heer J, Bostock M Crowdsourcing graphical perception: using mechanical turk to assess visualization design.

[43] Grady C, Lease M Crowdsourcing document relevance assessment with mechanical turk.

[44] Chen J, Menezes N, Bradley J, North TA (2011). Opportunities for crowdsourcing research on amazon mechanical turk. *Human Factors*.

[45] Kittur A, Smus B, Khamkar S, Kraut RE CrowdForge: crowdsourcing complex work.

[46] Clough P, Sanderson M, Tang J, Gollins T, Warner A (2012). Examining the limits of crowdsourcing for relevance assessment. *IEEE Internet Computing*.

[47] Scekic O, Truong HL, Dustdar S (2012). Modeling rewards and incentive mechanisms for social BPM. *Business Process Management*.

[48] Dow SP, Bunge B, Nguyen T, Klemmer SR, Kulkarni A, Hartmann B Shepherding the crowd: managing and providing feedback to crowd workers.

[49] Malone TW, Laubacher R, Dellarocas C (2009). Harnessing crowds: mapping the genome of collective intelligence. *MIT Sloan School Working Paper*.

[50] Kazai G, Kamps J, Milic-Frayling N Worker types and personality traits in crowdsourcing relevance labels.

[51] Vuurens J, de Vries AP, Eickhoff C How much spam can you take? an analysis of crowdsourcing results to increase accuracy.

[52] Vuurens JBP, de Vries AP (2012). Obtaining high-quality relevance judgments using crowdsourcing. *IEEE Internet Computing*.

[53] Tang W, Lease M Semi-supervised consensus labeling for crowdsourcing.

[54] Le J, Edmonds A, Hester V, Biewald L Ensuring quality in crowdsourced search relevance evaluation: the effects of training question distribution.

[55] McCreadie RM, Macdonald C, Ounis I Crowdsourcing a news query classification dataset.

[56] Xia T, Zhang C, Xie J, Li T Real-time quality control for crowdsourcing relevance evaluation.

[57] Zuccon G, Leelanupab T, Whiting S, Yilmaz E, Jose JM, Azzopardi L (2013). Crowdsourcing interactions: using crowdsourcing for evaluating interactive information retrieval systems. *Information Retrieval*.

[58] Zhu D, Carterette B An analysis of assessor behavior in crowdsourced preference judgments.

[59] Von Ahn L, Blum M, Hopper NJ (2003). CAPTCHA: using hard AI problems for security. *Advances in Cryptology—EUROCRYPT 2003*.

[60] Von Ahn L, Maurer B, McMillen C, Abraham D, Blum M (2008). reCAPTCHA: human-based character recognition via web security measures. *Science*.

[61] Sheng VS, Provost F, Ipeirotis PG Get another label? Improving data quality and data mining using multiple, noisy labelers.

[62] Welinder P, Perona P Online crowdsourcing: rating annotators and obtaining cost-effective labels.

[63] Ipeirotis PG, Provost F, Sheng VS, Wang J (2014). Repeated labeling using multiple noisy labelers. *Data Mining and Knowledge Discovery*.

[64] Hosseini M, Cox IJ, Milić-Frayling N, Kazai G, Vinay V (2012). On aggregating labels from multiple crowd workers to infer relevance of documents. *Advances in Information Retrieval*.

[65] Ipeirotis PG, Provost F, Wang J Quality management on amazon mechanical turk.

[66] Carpenter B Multilevel bayesian models of categorical data annotation.

[67] Raykar VC, Yu S, Zhao LH (2010). Learning from crowds. *The Journal of Machine Learning Research*.

[68] Snow R, O’Connor B, Jurafsky D, Ng AY Cheap and fast—but is it good? Evaluating non-expert annotations for natural language tasks.

[69] Dawid AP, Skene AM (1979). Maximum likelihood estimation of observer error-rates using the EM algorithm. *Applied Statistics*.

[70] Salakhutdinov R, Mnih A (2008). Probabilistic matrix factorization. *Advances in Neural Information Processing Systems*.

[71] Jung HJ, Lease M Inferring missing relevance judgments from crowd workers via probabilistic matrix factorization.

[72] Jung HJ, Lease M Improving quality of crowdsourced labels via probabilistic matrix factorization.

[73] Quinn AJ, Bederson BB Human computation: a survey and taxonomy of a growing field.

[74] Kulkarni A, Can M, Hartmann B Collaboratively crowdsourcing workflows with turkomatic.

[75] Soleymani M, Larson M Crowdsourcing for affective annotation of video: development of a viewer-reported boredom corpus.

